# An integrative review on the risk factors, prevention, and control strategies for carbapenem-resistant *Acinetobacter baumannii* colonization in critically ill patients

**DOI:** 10.3389/fmicb.2024.1519906

**Published:** 2025-01-10

**Authors:** Shihan Zhang, Jie Xiao, Yanan Li, Wei Li, Yihui Li, Mingmin Pang, Meichen Yan, Hui Han, Yi Cui, Xuehai Zhang, Hao Wang

**Affiliations:** ^1^Department of Critical Care Medicine, Qilu Hospital, Shandong University, Jinan, China; ^2^Innovation Research Center for Sepsis and Multiple Organ Injury, Shandong University, Jinan, China; ^3^Department of Clinical Laboratory, Qilu Hospital of Shandong University, Jinan, China

**Keywords:** CR*Ab*, colonization, risk factors, hazard, prevention

## Abstract

The presence of carbapenem-resistant *Acinetobacter baumannii* (CR*Ab*) has become one of the leading causes of life-threatening, hospital-acquired infections globally, especially with a notable prevalence in intensive care units (ICUs). The cross-transmission of microorganisms between patients and the hospital setting is crucial in the development of CR*Ab* colonization and subsequent infections. Recent studies indicate that colonization typically precedes infection, suggesting the effectiveness and necessity of preventing CR*Ab* colonization as a primary method to lower infection risks. As CR*Ab* infections tend to draw more attention due to their severe symptoms and poor outcomes, understanding the link between colonization and infection is equally vital. To establish a foundation for prevention and control strategies against CR*Ab* colonization in ICUs, we present a comprehensive review of research pertaining to CR*Ab* in ICUs. This encompasses an analysis of the resistance mechanisms and epidemiological characteristics of CR*Ab*, a discussion on associated risk factors, adverse outcomes, and an evaluation of detection methods and preventive strategies.

## 1 Introduction

The bacterium *Acinetobacter baumannii* is as an opportunistic human pathogen that predominantly infects critically ill patients worldwide. In recent years, the emergence and dissemination of carbapenem-resistant non-fermenting Gram-negative bacilli (NFGNB) in ICUs pose a substantial threat in hospitals (Agarwal et al., 2017; Kousouli et al., 2019). CR*Ab* ranks among the top five global pathogens in terms of attributable mortality due to antibiotic-resistant infections. It is estimated to be the leading pathogen causing mortality attributable to antibiotic resistance in South-East Asia, East Asia, and Oceania (Antimicrobial Resistance Collaborators, 2022a; European Antimicrobial Resistance Collaborators, 2023). In 2019, it caused more than 57,700 deaths and 1.5 million DALYs (deaths and disability-adjusted life-years) (Antimicrobial Resistance Collaborators, 2022b). A study focusing on European, Eastern Mediterranean, and African populations revealed that CR*Ab* had a pooled incidence of 41.7 cases per 1,000 patients, and accounts for 13.6% of hospital acquired infections in ICUs (Ayobami et al., 2019). And the resistance rate of CR*Ab* showed a significantly upward trend (Lăzureanu et al., 2016; He et al., 2020). Data from the China Antimicrobial Resistance Surveillance System (CHINET) showed that the resistance rates of *A. baumannii* strains to meropenem and imipenem increased from 30.1% and 39.0% in 2005 to 71.5% and 72.3% in 2021 (Liu et al., 2022). Severe infections caused by CR*Ab* have become a challenging clinical problem. The World Health Organization (WHO) listed CR*Ab* as one of the bacteria urgently needing antibiotics in its 2017 publication “Global Priority List of Antibiotic-Resistant Bacteria”(World Health Organization, 2017), and maintain its critical status until 2024 (World Health Organization, 2024). Besides, *A. baumannii* causes a range of nosocomial infections across multiple anatomical sites. Most commonly, *A. baumannii* infections manifest as ventilator-associated pneumonia or central line-associated blood stream infections (Harding et al., 2018). Previous retrospective studies found a significantly higher sputum isolation rate of CR*Ab* in ICUs compared to non-ICUs, and 80% of CR*Ab* in ICUs were isolated from sputum samples, accounting for over 50% of all carbapenem-resistant NFGNB (Neves et al., 2016; He et al., 2020).

Differentiating between colonization and infection is challenging. Generally, CR*Ab* colonization is defined as the presence of the bacteria on the skin, mucosa, open wounds, secretions, or indwelling medical devices without any associated clinical symptoms or signs of infection (fever, elevated white blood count, elevated inflammatory markers, and abnormal imaging) (Moghnieh et al., 2016; Neves et al., 2016; Bartal et al., 2022; Pascale et al., 2022).

In fact, the colonization of CR*Ab* often occurs earlier than the occurrence of infection, prior studies have described the presence of colonization as a factor associated with the development of *A. baumannii* infections. Latibeaudiere et al. (2015) found that patients with positive rectal or respiratory secretion cultures were eight times more likely to develop CR*Ab* infections. This finding remained significant even after adjusting for other variables such as sex, mechanical ventilation, and exposure to cephalosporins (Latibeaudiere et al., 2015). Other research data has shown that early colonization often leads to an increase in mortality, indicating the occurrence of poor prognosis (Zheng et al., 2021). This suggests that intervention during the early colonization phase of CR*Ab* may help improve patients’ long-term prognosis. However, due to difficulties in defining colonization and infection, and regional differences in ICUs conditions, research and attention in this field are currently limited. This article aims to summarize literatures associated to CR*Ab* colonization, integrate bacterial microbial characteristics, drug resistance, hazards, and prevention/intervention measures, provide certain warnings and guidance for clinical medicine, and advocate for attention to drug-resistant bacteria colonization.

## 2 Microbiological characteristics

**FIGURE 1:**
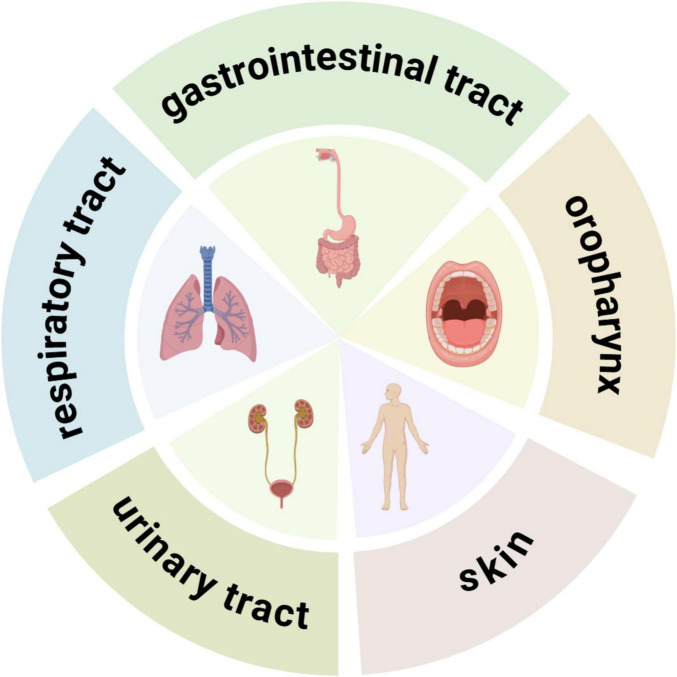
Common colonization sites of CR*Ab* in the human body.

*Acinetobacter baumannii*, a Gram-negative coccobacillus, measuring (0.6∼1.0) micrometers by (1.0∼1.6) micrometers, are mostly rod-shaped with blunt rounded ends, scattered or arranged in pairs, without spores or flagella, and the colonies are grayish-white, round, smooth, and have neatly defined edges form on a blood agar plate. *A. baumannii* is recognized for its adaptability and resilience in various environments. As a non-glucose fermenting and strictly aerobic bacterium, it can exist as a colonizing organism on human skin and other body sites, while also persisting in healthcare environments due to its robust biofilm formation (Figure 1). It withstands dry conditions effectively and harbors resistance determinants against antibiotics and commonly used disinfectants, increasing its propensity to contaminate hospital settings. Compared to other pathogens, it can survive for more extended periods on surfaces, including hospital fixtures, utensils, equipment, invasive medical devices, and personnel (Nutman et al., 2016; Odih et al., 2022).

## 3 Drug resistance mechanisms

For Gram-negative bacteria, common resistance mechanisms encompass enzymatic hydrolysis by β-lactamases, overexpression of drug efflux pumps, and mutations in antibiotic target sites (Li et al., 2015; Arzanlou et al., 2017). The mechanisms of carbapenem resistance in *A. baumannii* can generally be categorized into four distinct groups: alterations in penicillin-binding proteins, loss of outer membrane porins, overexpression of efflux pumps, and synthesis of carbapenem-hydrolyzingβ-lactamases (Nguyen and Joshi, 2021), while the last group is reported to be the most important one (Mugnier et al., 2009; Higgins et al., 2013).

The OXA-type carbapenem-hydrolyzing class D β-lactamases [oxacillinases (OXAs)] constitute a diverse family, mainly including blaOXA-23-like, blaOXA-40-like, blaOXA-58-like, blaOXA-143-like, and blaOXA-235-like (Nowak and Paluchowska, 2016; Juan et al., 2019). Their expression could be enhanced by the insertion of an upstream IS, which enhances expression by providing a strong promoter, causing high resistance levels (Mugnier et al., 2009; Sen and Joshi, 2016).

A recent study reported an update on the molecular epidemiology and global distribution of carbapenemase encoding genes. Among all international clones (IC) IC1–IC8, they found IC2 predominated with 196 of 313 isolates (62.6%) was spread over all participating regions, ranging from 10% in Latin America to 70% in North America and about 80% in Africa, Asia, and Europe. The most frequently encountered carbapenemase-encoding genes were blaOXA-23-like and blaOXA-40-like, present in 234 of 300 (74.8%) and 56 of 300 isolates (17.9%), respectively. While the number of blaOXA-40-like-positive isolates was considerably higher in Latin and North America than in the remaining regions with nearly 32% of all American isolates (Müller et al., 2023). Overall, the predominance of blaOXA-23-like producing CR*Ab* mainly representing IC2 has been reported worldwide (Higgins et al., 2010; Eigenbrod et al., 2019; Hamidian and Nigro, 2019).

## 4 Risk factors

Numerous factors contribute to the colonization of CR*Ab*, highlighting the complexity of this healthcare challenge. In our prior research, we revealed patients testing positive for CR*Ab* were more likely to have cardiovascular diseases, type II diabetes mellitus, solid tumors and suspected sepsis, and this seems to be explained by impairment of structure and function of certain body systems and immunosuppression (Xu et al., 2019a; Zheng et al., 2021). Other certain patient-associated factors like prolonged hospitalization, a history of previous hospitalization, and the severity of the underlying disease can also be linked to CR*Ab* colonization (Thoma et al., 2022). In addition to patient disease-related factors, there still two main opportunities for the occurrence and dissemination of colonization events, namely the environment and the medical treatment.

### 4.1 Environment

As we mentioned before, *A. baumannii* possesses tenacity to persist in environments for prolonged periods, and this explains one of the main reasons for the occurrence of CR*Ab* colonization. It is largely believed that two attributes, drug resistance and environmental persistence, have enabled *A. baumannii* to thrive in the nosocomial environment (Roca et al., 2012). Commonly, healthcare environments include prolonged periods of desiccation and routine disinfection regimes. Like their resistance to antibiotics, *A. baumannii* has evolved to withstand these environmental stresses (Wong et al., 2017). Desiccation resistance, which is the ability to maintain viability under dry conditions, varies amongst clinical isolates of *A. baumannii*, with some isolates remaining viable for almost 100 days (Harding et al., 2018). Besides, the biofilm formation also help increase tolerance to extracellular stresses (Greene et al., 2016a; Greene et al., 2016b).

Patients colonized with CR*Ab* serve as carriers, contaminating their immediate surroundings like drawer handles of supply carts, floors, infusion pumps and ventilator buttons, etc. (Thom et al., 2011; Alfandari et al., 2014; Latibeaudiere et al., 2015; Shimose et al., 2016; Weiss et al., 2016). Ng et al. (2018) reported environmental contamination present in 5 of 18 (28%) of the rooms housing patients with CR*Ab*, by collecting surface swabs of the environment inside and outside the ward. Uwingabiye et al. (2017) also observed a genetic resemblance in 80 of 83 (96.4%) of all isolates between environmental and clinical, showing that the clonal spread of environmental *A. baumannii* isolates is related to that of clinical isolates recovered from colonized or infected patients. Interestingly, some studies found a significantly difference in the degree of contamination based on the occupant’s anatomic source of *A. baumannii* (Rosa et al., 2014). After sampling the air and environmental surface swab daily for up to 10 days, Shimose et al. (2016) found the positive rate of air samples in rooms with rectally colonized patients is nearly three times (38.3% vs. 13.1%) that of patients with respiratory colonization. And the positive rate of environmental samples also showed the same trend (15.5% for patients with rectally colonization, 9.5% for patients with respiratory colonization) (Shimose et al., 2016).

Cross-transmission triggered by medical personnel contacting patients without proper disinfection forms critical cause of CR*Ab* colonization/infection within ICUs wards (Russotto et al., 2015). Inadequate compliance with personal protective equipment (PPE) and hand hygiene are common contributing factors (Thoma et al., 2022). Instances like failure to remove gloves post-contact with the patient’s surrounding environment, coupled with the inadequate performance of the hand-hygiene action, are associated with heightened cross-contamination issues (Pires et al., 2017). Therefore, admitting patients who are CR*Ab* carriers into ICUs certainly pose a nosocomial infection risk to other susceptible individuals (Maamar et al., 2018; Al-Hamad et al., 2020).

### 4.2 Medical treatment

#### 4.2.1 Unreasonable antibiotic use

Neves et al. (2016) found that previous antibiotic treatment significantly contributed to the risk factors associated with CR*Ab* colonization. Munoz-Price et al. (2016) found that once a patient is exposed to carbapenems, even if the disease severity is adjusted, the risk of colonization increases fourfold, and the impact will persist. Our team’s previous research also showed that the use of broad-spectrum antibiotics for over 7 days was an independent predictor of lower respiratory CR*Ab* infection/colonization in mechanically ventilated patients. The CR*Ab* detection rate in patients with antimicrobial combination therapy was 2.5% higher than non-antimicrobial combination therapy patients (Xu et al., 2019a). The reason for this phenomenon seems to be explained by adaptive resistance (Skiada et al., 2011). Previous studies have shown that multiple genes mediate this process, like the production of OXA-23 and TEMoneira leading to β-lactam resistance (Desmoulin et al., 2024), and the mutations in pmrCAB and lpxA gene may explain the adaptive resistance to polymyxins (Barin et al., 2013). Besides, overuse of antibiotics for presumed bacterial co-infection or secondary infection may also lead to the development and spread of bacterial resistance (Langford et al., 2020). Table 1 lists some antibiotics that may influence the CRAb colonization.

**TABLE 1:** Antibiotics use of CR*Ab* colonization reported over the past 10 years.

Antibiotic	Colonization site	Year	Reference
Carbapenems	Gastrointestinal tract	2022	Wong et al., 2022
	Oropharynx, bilateral axilla, umbilicus, perianal areas	2022	Rizk et al., 2022
	Rectum	2024, 2021	Meschiari et al., 2021a; Hu et al., 2024
Other β -lactams (except for carbapenems)	Gastrointestinal tract	2022	Wong et al., 2022
	Rectum	2024	Hu et al., 2024
Glycopeptides	Rectum	2021	Meschiari et al., 2021a
Piperacillin/tazobactam	Rectum	2021	Meschiari et al., 2021a
Polymyxin	Rectum	2022, 2014	Freire et al., 2014; Sharma et al., 2022
	Axillary	2022	Sharma et al., 2022
	Groin	2014	Freire et al., 2014
Fluoroquinolones	Rectum and nasal	2015	Cheng et al., 2015
Antimicrobial combination therapy	Rectum	2021	Meschiari et al., 2021a

#### 4.2.2 Invasive treatment

Previous studies have discovered a strong association between the use of invasive devices and CR*Ab* colonization in a hospital environment (Moghnieh et al., 2016). Patients who receive supportive treatments like renal replacement therapy, longer invasive ventilation, and vasopressor treatment have been proved to have a high tendency for CR*Ab* colonization (Zheng et al., 2021). The rate of lower respiratory CR*Ab* infection/colonization in tracheotomy patients is 1.996 times higher than that in patients with tracheal intubation (Xu et al., 2019a). Neves et al. (2016) observed that invasive devices comprise a significant proportion of risk factors associated with CR*Ab* colonization. Meschiari et al. (2021a) found the use of permanent devices and the use of invasive devices in a hospital environment significantly associated to risk of rectal colonization with CR*Ab*. Among these were the use of vascular catheters, such as central venous catheterization (CVC) or the use of peripherally inserted central catheter (PICC) or Midline, urinary catheter (UC), nasogastric tube (NG), and mechanical ventilation (MV). Other factors that were significantly associated were tracheostomy and percutaneous endoscopic gastrostomy (PEG), which respectively determined an elevated risk for rectal CR*Ab* colonization of 10.5 and 11 times (Meschiari et al., 2021a).

**FIGURE 2:**
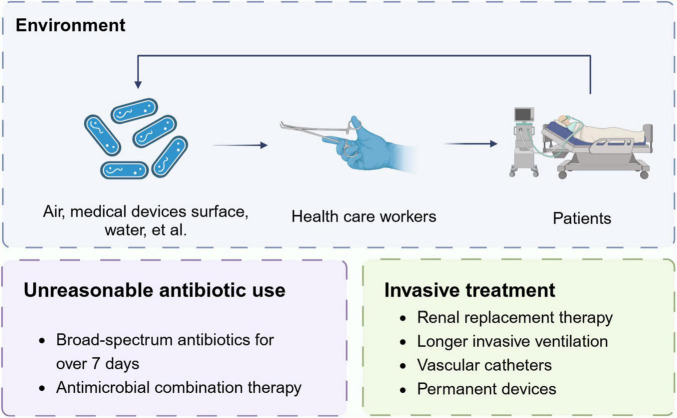
Risk factors of CR*Ab* colonization. The figure shows main factors for the occurrence and dissemination of colonization events.

Overall, the above factors increase the risk of CR*Ab* implantation in ICUs patients. In order to obtain much better prognosis, we should comprehensively understand the current colonization risk associated to environment and treatment, and much more efforts are needed urgently (Figure 2).

## 5 From colonization to infection: mechanisms and hazards

The harm induced by CR*Ab* infection poses challenges for all doctors. Generally, the process of bacterial infection consists of three steps. First, bacteria invade or colonize initial sites of infection. Second, bacteria overcome host barriers, such as immune responses, and disseminate from initial body sites to the bloodstream. Third, bacteria adapt to survive in the blood and blood-filtering organs. Previous studies have confirmed that colonization precedes infection. There is evidence suggesting that colonization of *A. baumannii* in seemingly harmless body parts such as armpits, pharynx, and gastrointestinal tract in ICUs may precede subsequent infections (Ayats et al., 1997). Numerous studies indicate that colonization is a crucial risk factor for subsequent infections (Moghnieh et al., 2016; Gorrie et al., 2017; Karampatakis et al., 2018). In fact, the burden of colonization, expressed as the presence of CR*Ab* at different sites, and the homeostasis alteration in particular organs, such as the gut and the lungs, may favor bacterial translocation and subsequent CR*Ab* infection (Cogliati Dezza et al., 2023). And this process is mediated by multiple mechanisms including bacterial invasiveness and toxicity (iron uptake, siderophore, immune evasion, and biofilm formation) (Xiao et al., 2022), immunosuppression, multisite colonization, burden of comorbidities and mechanical ventilation, etc.

**FIGURE 3:**
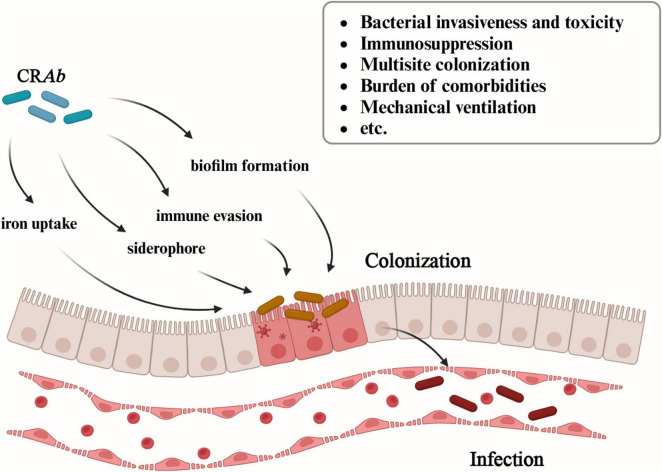
Multiple mechanisms of CR*Ab* from colonization to infection. The figure illustrates the mechanisms involved in CR*Ab* from colonization to infection, including bacterial invasiveness and toxicity (iron uptake, siderophore, immune evasion, and biofilm formation), immunosuppression, multisite colonization, burden of comorbidities and mechanical ventilation, etc.

Recent study demonstrated the process of biofilm formation from CR*Ab* colonization to infection. Generally, biofilm production relies on the initial reversible bacterial attachment to a surface in response to environmental stimuli (Toyofuku et al., 2016). Early adhesion is essential in the colonization process and in establishing an *A. baumannii* infection. Recent researches found the CsuA/BABCDE chaperon-usher assembly system and the two-component system BfmRS are critical in biofilm formation, while the AdeABC, AdeIJK, and AdeFGH RND-type efflux systems play a central role in the initial stages of adhesion, surface colonization, and biofilm maturation in *A. baumannii* (Coyne et al., 2011). In the final stage, the cells within the biofilm disperse and colonize new surfaces (Cavallo et al., 2023; Figure 3).

According to our research, when compared to non-colonized patients, CR*Ab* colonized patients had significantly higher APACHE II scores and 6-month mortality rates (68.8% vs. 43.5%; *P* < 0.001), and longer ICUs stays (Zheng et al., 2021), and this finding corroborates earlier studies. Latibeaudiere et al. (2015) showed that patients carrying this pathogen found on surveillance cultures during their ICUs stay were at a 16.3 times higher risk of developing CR*Ab* infections. Bacterial colonization frequently results in subsequent respiratory and digestive tract infections, and hospital infections (Maamar et al., 2018). Munoz-Price et al. (2016) found that CR*Ab* colonization is the strongest variable associated with subsequent clinical infections of CR*Ab*. The APACHE II score is strongly correlated with the acquisition of *A. baumannii*, leading to higher mortality rates and longer hospital stays, imposing a substantial burden on patient prognosis and treatment costs. The likelihood of subsequent bloodstream infections in patients carrying CR*Ab* is seven times higher than in non-CR*Ab* colonized patients (OR = 7.41, 95% CI = 2.39–22.92), and the mortality rate in CR*Ab* colonized patients is 50%. Compared with non-CR*Ab* colonized patients (OR = 1.84, 95%; CI = 0.69–4.90), the risk of death in CR*Ab* colonized patients is almost two times higher (Odih et al., 2022). According to another research, the patients with CR*Ab* colonization still show a high crude mortality rate, even if without a subsequent infection onset (Cogliati Dezza et al., 2023). Besides, a recent metagenomic research found the correlation between CR*Ab* colonization/infection and respiratory tract microbiome dysbiosis. The results showed that the relative abundance of *Acinetobacter* increased in the order CR*Ab* non-colonization, colonization and infection, while the α and β diversity of the lower respiratory tract microbiome was opposite. These dynamic evolution of pulmonary microbiota could promote the occurrence of infection and leading a poor prognosis (Xiao et al., 2022).

In addition, for bloodstream infection, it often occurs in patients with low immune function or serious basic diseases, such as long-term use of immunosuppressants, malignant tumors or diabetes. CR*Ab* can enter the bloodstream through skin wounds, intravascular catheters, and other routes, leading to bacteremia or sepsis. Therefore, we may need to pay more attention to colonized patients with immunosuppression or invasive treatment. Overall, whether acquired during the stay in ICUs or imported, colonization by CR*Ab* is a significant risk factor for serious nosocomial infection, causing prolonged ICUs hospitalization duration, elevated patient costs, and lower overall survival (Phu et al., 2017; Kousouli et al., 2019; Liu et al., 2020; Zhen et al., 2020; Ejaz et al., 2021).

## 6 Measures to prevent colonization

CR*Ab* colonization is a critical step before the onset of hospital-acquired infections and presents significant risks. Intervention and prevention of colonization issues should be implemented as early as possible.

Currently available strategies for reducing the transmission of healthcare–associated pathogens include a comprehensive bundle of measures (Rutala and Weber, 2019; Meschiari et al., 2021b; Seok et al., 2021). These include the development of evidence-based policies and procedures, the selection of suitable cleaning and disinfecting agents, and the education of staff across various departments, including environmental services, patient care equipment, and nursing. Compliance monitoring is also crucial, focusing on the thoroughness of cleaning and the proper use of products, with feedback mechanisms such as just-in-time coaching. Furthermore, the adoption of “no-touch” room decontamination technologies is instrumental in ensuring compliance with contact and enteric precautionary measures for patients (Rutala and Weber, 2019). Considering the possibilities in practical operation of preventing CR*Ab* colonization, we will select a few points to discuss in detail.

### 6.1 Environmental cleaning and disinfection

Proper environmental disinfection and sterilization are important measures to reduce CR*Ab* colonization. A study found the level of contamination is related to the patient’s colonization load (Nutman et al., 2016). Through the collection of swabs from the patient’s oral mucosa and rectum, along with sponge swabs from the patient’s skin and surrounding environment, it was discovered a positive correlation between the patient colonization score and the environmental contamination score (*r* = 0.63, *P* < 0.001). In fact, high-touch surfaces such as ventilators, control panels, infusion pumps and bedrails are considered to be important objects in the potential chain of transmission from one patient to another (Ng et al., 2018). Therefore, environmental management is of great significance.

Previous studies have shown that the cleanliness of the patient’s surroundings has been proven to be effective in reducing *Acinetobacter* colonization or infections (Lerner et al., 2020; Li et al., 2021). Using aerosolized hydrogen peroxide (aHP) system or manual hypochloride cleaning after discharge of a known CR*Ab*-carrier decreased room contamination by 78% or 85%, respectively (Lerner et al., 2020). Other studies have shown that daily disinfection and care for patients can also reduce CR*Ab* colonization/infection and improve long-term prognosis. In ICUs with high prevalence of CR*Ab*, daily use of 2% chlorhexidine gluconate bath can reduce cross transmission of CR*Ab* between patients (Hong et al., 2018; Metan et al., 2020; Suh et al., 2021). Chung et al. (2015) revealed a 51.8% reduction of CR*Ab* acquisition rates in the medical ICU with CR*Ab* endemicity during a 12-month chlorhexidine bathing period. And the CR*Ab* contamination of environment, like patient gowns, bed rails, staff gowns, keyboards, and monitors was also significantly reduced during the chlorhexidine bathing period (Chung et al., 2015).

However, the frequent use of disinfectants has led to strains less susceptible to these agents (Biswas et al., 2018), while relevant studies showed the qacE and qac△E1 genes of CR*Ab* isolates associated with a higher minimum inhibitory concentration and less susceptible to benzalkonium bromide and chlorhexidine gluconate (Guo and Li, 2019). Besides, enhanced environmental cleaning alone may be insufficient to curb the acquisition of CR*Ab* in a highly endemic ICU. Simply increasing the frequency of cleaning without enhancement of other bundle interventions for CR*Ab* reduction did not lead to a significant reduction in CR*Ab* acquisition (Seok et al., 2021). Multiple factors like invasive therapies (Seok et al., 2021) and unique biological characteristics, particularly for isolates forming biofilms (Espinal et al., 2012), causing re-contamination during the disinfection interval. This reminds us to improve the cleaning standards for patients receiving different treatments, and pay more attention to the quality of single cleaning and the interval between two cleanings, with the goal of refining cleaning procedures to effectively diminish clinical exposure.

### 6.2 Advancement of existing monitoring technologies

Enhancing the sensitivity and efficiency of detection would be a good way to decrease the colonization risk of CR*Ab*. Previous evidence from simulation models demonstrates the potential benefits of active surveillance (Lee et al., 2011). One of the model has demonstrated that as screening sensitivity approaches 90%, the reduction in transmissions, infections, and deaths can reach 78% (with a range of 77%–80%), accompanied by cost savings between 22% and 53%, when the prevalence of carriage is between 2% and 6% (Coyle et al., 2014). Besides, since the actual treatment plan largely depends on the patient’s test results, false-positive test results or empirical antibiotic misuse due to diminished testing efficiency are leading contributors to some CR*Ab* colonization cases. Especially during the COVID-19 pandemic, patients overload caused delayed culture and drug sensitivity results in microbiological laboratories, leading to inappropriate antibiotic treatment and resulting in widespread CR*Ab* colonization in ICUs (Abelenda-Alonso et al., 2020; Vaughn et al., 2021). Therefore, improve detection technology is an effective strategy to alleviate CR*Ab* colonization caused by unreasonable treatment.

Culture and polymerase chain reaction (PCR) are traditionally considered the most common methods for pathogen epidemiological screening. Recently, researchers have used loop-mediated isothermal amplification (LAMP) technology to detect *A. baumannii* and carbapenem antibiotic resistance. They found LAMP to be a promising method for early detection (Garciglia-Mercado et al., 2021; Sharma and Gaind, 2021). LAMP’s sensitivity is 10 times higher than PCR (Garciglia-Mercado et al., 2021), and apart from LAMP, whole-genome sequencing (WGS) is also a valuable tool in epidemiological research (Ng et al., 2018; Venditti et al., 2019). In fact, high sequencing costs and lacking professional testing technicians resulting in only some regions having the conditions to implement the test that affect the widely used of WGS. Perhaps establishing a convenient and efficient testing industry chain can better solve the problems such as cost and specimen transportation, thereby better serving surrounding medical institutions. Overall, LAMP appears to be an effective screening method for CRAB epidemiological investigations, effectively balancing sensitivity with detection costs (Hong et al., 2018).

### 6.3 Hospitalization education

Furthermore, there is a need to stress hospital awareness education for healthcare personnel and enhance guidelines compliance such as hand hygiene. Previous researches had indicated that enhanced adherence to hand hygiene protocols correlates with a reduction of healthcare-associated infections in general and multidrug-resistant organisms (Lotfinejad et al., 2021). Although hand hygiene might seem simple, compliance in healthcare settings has always been far from ideal worldwide (Gould et al., 2017). In a related study, we found that hand hygiene compliance before patient contact is relatively low, indicating a disregard of hospital infection prevention and control principles by healthcare personnel, and this leads high occurrence of nasal pathogenic bacteria colonization among ICUs medical staff (Xu et al., 2019b). Actually, available evidence shows that compliance with hand hygiene recommendations during healthcare delivery remains suboptimal around the world, with an average of 59.6% compliance levels in intensive care units up to 2018, and extreme differences between high income and low income countries (64.5% vs. 9.1%) (de Kraker et al., 2022). Besides, some research found the seniority of medical workers also correlated with the incidence and prevalence of CR*Ab* acquisition. In our previous research, we found that nursing staff with lower seniority are more prone to bacterial colonization. This could be explained that despite receiving standardized training, junior nursing staff often possess less experience or are prone to making more errors, which consequently leads to their performance not matching the excellence demonstrated by their senior counterparts (Xu et al., 2019b; Seok et al., 2021). Therefore, further optimization of hand hygiene education and supervision is urgently needed.

World Health Organization designed “The My 5 Moments for Hand Hygiene approach,” which helped to highlight hand hygiene indications to facilitate understanding, training, and monitoring in a broad range of healthcare settings worldwide. This concept has gained universal acceptance and serves as the foundation of WHO’s multimodal strategy for enhancing hand hygiene. However, the accumulated evidence has suggested that multimodal promotion strategies are more effective than single interventions in changing healthcare workers’ behavior, considering the complex and multifactorial determinants of hand hygiene compliance (Allegranzi et al., 2013; Zingg et al., 2015). Bundled interventions that incorporating the multimodal hand hygiene improvement strategies include additional measures such as goal setting, reward incentives, and accountability, have demonstrated promising outcomes in boosting compliance (Luangasanatip et al., 2015). In summary, more implementation strategies should be combined with different regions and cultures to better motivate and supervise clinical workers.

### 6.4 Standardized medical treatment

Unreasonable medical practice is also an important risk factor for the occurrence of CR*Ab* colonization, so it is important to follow strict antibiotic use guidelines and to carry out adequate assessment before performing invasive procedures. In addition, strict and standardized disinfection and aseptic operations are also important guarantees for preventing bacterial colonization and infection.

### 6.5 Summary

**FIGURE 4:**
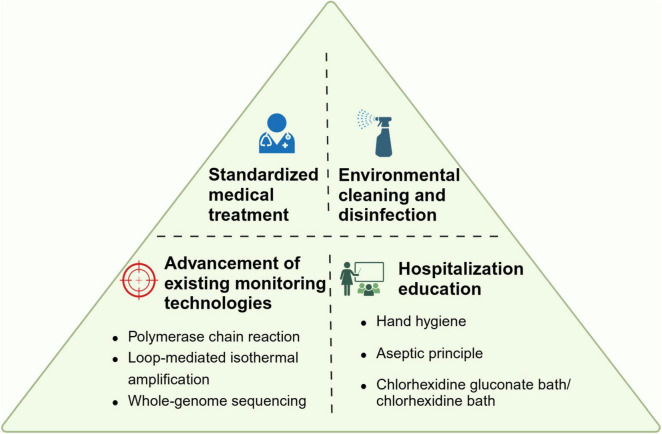
Proposal bundle to prevent CR*Ab* colonization. The figure summarized four aspects of preventive measures.

Honestly, according to a guideline reported by ESCMID-EUCIC (European Committee of Infection Control-European Society of Clinical Microbiology and Infectious Diseases), there is not sufficient evidence suggesting the positive effects brought by decolonization intervention (Tacconelli et al., 2019). However, some measures significantly increased the 14-day eradication of CR*Ab* that including daily 4% chlorhexidine body wash, inhaled colistin (160 mg twice daily), colistin sulfate [50 mg (salt) four times daily] and tobramycin (80 mg four times daily) (Agustí et al., 2002; Borer et al., 2007; Kuo et al., 2012; Chen et al., 2014). Based on the current situation, we believe that there will be more research guiding a better response to the challenge brought by CR*Ab* colonization in the future (Figure 4).

## 7 Conclusion

Carbapenem-resistant *Acinetobacter baumannii* colonization, as a relatively overlooked topic in the current research field of drug-resistant bacteria, has received increasing attention in recent years. It is undeniable that early intervention in the occurrence and development of colonization issues is extremely important and urgent. Preventing the colonization of CR*Ab* can better improve the patient’s prognosis, shorten hospitalization time, and save related medical expenses; on the other hand, it also saves valuable medical resources, enabling clinical workers to carry out medical work more efficiently. In addition to more efficient detection techniques, there is still a lack of research on whether specific clones are responsible for both environmental colonization and ICUs infection (Jiang et al., 2022). But we advocate that more clinical workers should pay attention to the impact of CR*Ab* colonization and provide more comprehensive ideas for subsequent individualized treatment.
